# How to calculate the annual costs of NGO-implemented programmes to support orphans and vulnerable children: a six-step approach

**DOI:** 10.1186/1758-2652-14-59

**Published:** 2011-12-19

**Authors:** Bruce A Larson, Nancy Wambua

**Affiliations:** 1 Department of International Health, Boston University School of Public Health, Boston, Massachusetts, USA; 2 Center for Global Health and Development, Boston University, Boston, Massachusetts, USA; 3 Finance Department, Benevolent Institute for Development Initiatives, Machakos, Kenya

## Abstract

**Background:**

Information on the costs of implementing programmes designed to provide support of orphans and vulnerable children (OVC) in sub-Saharan Africa and elsewhere is increasingly being requested by donors for programme evaluation purposes. To date, little information exists to document the costs and structure of costs of OVC programmes as actually implemented "on the ground" by local non-governmental organizations (NGOs). This analysis provides a practical, six-step approach that NGOs can incorporate into routine operations to evaluate their costs of implementing their OVC programmes annually. This approach is applied to the Community-Based Care for Orphans and Vulnerable Children (CBCO) Program implemented by BIDII (a Kenyan NGO) in Eastern Province of Kenya.

**Methods and results:**

The costing methodology involves the following six steps: accessing and organizing the NGO's annual financial report into logical sub-categories; reorganizing the sub-categories into input cost categories to create a financial cost profile; estimating the annual equivalent payment for programme equipment; documenting donations to the NGO for programme implementation; including a portion of NGO organizational costs not attributed to specific programmes; and including the results of Steps 3-5 into an expanded cost profile. Detailed results are provided for the CBCO programme.

**Conclusions:**

This paper shows through a concrete example how NGOs implementing OVC programmes (and other public health programmes) can organize themselves for data collection and documentation prospectively during the implementation of their OVC programmes so that costing analyses become routine practice to inform programme implementation rather than a painful and flawed retrospective activity. Such information is required if the costs and outcomes achieved by OVC programmes will ever be clearly documented and compared across OVC programmes and other types of programmes (prevention, treatment, etc.).

## Background

An estimated 56.1 million children in sub-Saharan Africa had lost one or both parents as of 2009 [1]. Among this total, 14.9 million children lost one or both parents due to AIDS, and large numbers of other children are vulnerable to becoming orphans because one or both parents are HIV infected.

In response to the diverse problems and needs of orphans and vulnerable children (OVC) in low-income countries, a range of programmes have evolved over time to attempt to improve their daily lives and future prospects. The US government, through the US President's Emergency Plan for AIDS Relief (PEPFAR), spent $312 million on OVC activities in 2008 [2]. Between 2006 and 2008, more than $1 billion was spent on OVC programmes, the majority of which targeted OVC being cared for in the community (extended family members, other households) [3]. As part of The Reauthorization Act of 2008, significant sums will continue to be allocated to OVC programmes between 2009 and 2013 [4].

PEPFAR-supported OVC programmes typically involve a set of organizations working together to implement an overall OVC programme. As one example, Christian Aid was the prime recipient for the Community Based Care for Orphans and Vulnerable Children (CBCO) Program implemented during 2005-2010 in Nigeria, Uganda, Kenya and Zambia. Christian Aid then collabourated with a small number of lead non-governmental organizations (NGOs) in each country to implement the overall programmes. In Kenya, the Benevolent Institute for Development Initiatives (BIDII), based in Machakos, Eastern Province, implemented the programme in Eastern Province; the Inter-Diocesan Christian Community Services (IDCCS), based in Kisumu, implemented the programme in Nyanza Province. In the CBCO programme, as is typically the case with OVC programmes funded through PEPFAR, local NGOs operating at a sub-national level deliver programme services to OVC and their households.

Local NGOs, such as BIDII, typically implement "their" OVC programme using funds from multiple sources including donor funds, the NGOs' own resources, volunteers and donations from local communities and perhaps from such sources as other programmes and the government. Thus, the resources used to implement an NGO's OVC programme (the costs of implementation) are not simply the amounts budgeted within PEPFAR-funded programmes.

A review of the literature on the costs, outcomes and cost effectiveness of OVC programmes concluded that little information exists to document the costs and structure of costs of OVC programmes as actually implemented "on the ground" by NGOs [5]. This information is required if evaluations of OVC programmes in terms of costs and outcomes (cost-outcomes analysis, cost-effectiveness analysis) are to be completed and if high-performing types of programmes are to be replicated and expanded elsewhere.

The demand for more and better information on the costs of interventions is directly included in the United States Agency for International Development's (USAID's) Evaluation Policy statement. Increased demand for costing information is also embedded into the growing demand for implementation science, including cost-effectiveness analysis in relation to HIV/AIDS programming [6]. Because of the multi-dimensionality of OVC programmes, as we have discussed, we prefer to use the term, "cost-outcomes analysis", instead of "cost-effectiveness analysis" [5,7].

The concepts and methods for evaluating the costs of programmes and projects, whether investments in irrigation infrastructure, HIV prevention programmes, antiretroviral treatment programmes or OVC programmes, are well documented elsewhere in textbooks and donor-related documents [7-11]. The World Bank's "OVC Toolkit for sub-Saharan Africa" website also includes very reasonable guidance on costing of OVC programmes, which itself is essentially a replication of standard training materials on costing of projects within the broader field of benefit-cost analysis [12]. A small number of studies have applied these methods to evaluate the costs of OVC programmes [13-17].

However, such methods are not widely integrated into routine practices of OVC programmes, in part because existing toolkits are rather vague on how to obtain the information needed to apply the methods. While textbooks and toolkits are important, it is difficult to convey in such materials the experience, creativity and decisions needed to implement the methods.

The goal of this paper is to show through a concrete example how NGOs implementing OVC programmes (and other public health programmes) can organize themselves for data collection and documentation prospectively during the implementation of their OVC programmes. The significance here is that costing analyses can become routine practice to inform programme implementation, rather than a painful and flawed retrospective activity. Rather than attempting to train programme implementation staff on these procedures, financial/accounting staff (perhaps one person) can logically perform costing analyses as a relatively minor addition to their existing activities. Such information is required if the costs and outcomes achieved by OVC programmes will ever be clearly documented and compared across OVC programmes and other types of programmes (prevention, treatment, etc.).

To achieve this goal, this paper provides a logical six-step approach that researchers and local NGOs themselves can use to document and describe the annual costs of implementing their programmes. The paper is organized as follows. The six-step approach is explained in the Methods section. The Results section then provides a detailed example using the CBCO programme implemented by BIDII in Eastern Province of Kenya. A few additional final issues are then addressed in the Discussion section. The paper concludes with a set of practical recommendations for integrating this six-step method into routine practice during programme implementation.

## Methods

Table 1 provides a brief summary of the six steps for evaluating the costs of implementing OVC support programmes. A prerequisite for costing of any intervention is a clear definition of the intervention. OVC programmes implemented by NGOs typically provide multiple sets of inputs to OVC and/or their households, such as food and nutrition support, access to health services, psychosocial support, educational support, and support for household economic strengthening. Through these activities, the programmes work to improve the welfare of OVC and their households along several dimensions (improved food security, educational and psychosocial outcomes, household access to credit, improved income and household wealth, etc.) [5].

**Table 1:** A summary of the six-step approach approach

Prerequisite	Develop clear definition of NGO's OVC programme
Step 1.	Access and organize NGO's annual financial report.

Step 2.	Link financial report sub-categories from Step 1 into input cost categories and create financial cost profile.

Step 3.	Estimate the annual equivalent payment for programme equipment.

Step 4.	Document donations to the NGO for programme implementation.

Step 5.	Include a portion of NGO organizational costs not attributed to specific programmes (as explained in Step 5).

Step 6.	Include the results of Steps 3-5 into an expanded cost profile.

In general economic terms, OVC programmes operate like multiple input and multiple output firms, just like household-based farming operations that combine multiple inputs (labour, fertilizers, seeds) to produce multiple types of crops (cassava, maize, plantains) on the same piece of land. To begin any costing analysis of an OVC programme, a clear description of the complete OVC programme is required, not just one portion of the programme. This issue will be discussed further during the example provided in the Results section.

### Step 1. Access and organize financial reports

The first step is to access and review the NGO's annual financial report that documents itemized expenditures for the programme during a year. Itemized expenditures, sometimes called expenses, are payments actually made by the NGO. Such expense reports are routinely produced by organizations for accounting, tax reporting and donor reporting purposes. NGOs will typically have an overall annual financial report that encompasses all its activities. This overall NGO financial report will typically include (or be based on) a number of sub-reports for each external funding source. It is necessary to access the "programme-specific" annual financial report. Such reports are typically developed by the NGO's accounting or financial staff using a spreadsheet programme, such as Microsoft Excel.

In some situations, an NGO might implement its OVC programme with funding from multiple sources (e.g., USAID, the UK's Department for International Development, donations from a US faith-based organization, local government funds, and/or donations from a local church congregation). Regardless of where the funds come from, the NGO will have an annual financial report, which may include a sub-report for each donor. When a programme is implemented with funding from multiple donors, each with perhaps a different fiscal year for reporting expenses, a costing analysis based on a calendar year regardless of funding source would be a logical approach. Alternatively, the fiscal year that coincides with the NGO's fiscal year or the largest donor's fiscal year could be used.

A prerequisite to complete Step 1 and to proceed with any costing analysis of NGO programmes is the willingness of the NGO and its staff, especially the financial and/or accounting staff and the programme manager, to support the activity. This is easiest when the NGO itself is undertaking the analysis and the funders and NGO management agree that such information is needed for on-going project management and evaluation purposes.

### Step 2. Link financial report sub-categories to input cost categories

The purpose of Step 2 is to reorganize the information contained in the financial report into logical groups of expenses for key categories of "inputs" used in the implementation of the project. Just like seeds, pesticides, fertilizer, land, household and hired labour are key inputs in agricultural production, NGO-implemented OVC programmes have some underlying "production technology" that transforms inputs into outputs. Typical input categories include office and buildings, vehicles, programme staff, office equipment, office supplies, supplies and items provided directly to OVC and their households. As will be shown in the Results section, Step 2 can be accomplished relatively easily through minor adjustments to financial reports (the Excel file used for expense reporting). Thus, no new software or models are needed to complete the analysis.

Knowledge of the programme and the information developed as part of Step 1 will provide the information needed to identify logical input categories for an OVC programme. In our experience, the NGO programme inputs can typically be organized into a relatively small number of key input categories that describe how the project was actually implemented, such as payments for education, NGO staff salaries, transportation, small stipends to volunteers, and agricultural inputs. In most cases, while input categories could be further disaggregated into addition sub-categories (fuel, vehicle servicing, insurance), the additional level of detail is typically not required.

If the NGO also sub-contracts to another organization to assist with implementing the programme, then the NGO's financial report will typically include payments to the sub-contract recipient (for example, quarterly transfers of funds). In such situations, the costing analysis would be completed twice (one for the prime organization and one for the sub-contract recipient) for a complete costing analysis.

### Step 3. Estimate the annual equivalent payment for programme equipment

NGOs will typically have an inventory of equipment purchased specifically for the programme. Some of these items are purchased directly by the NGO (and included as an expense in the programme-specific financial report), but some could be purchased by another organization or the funding agency and provided to the NGO (and therefore not included in the NGO's financial report). Regardless of who purchased the equipment, the NGO should have an inventory list of equipment in its possession (typically for insurance purposes, avoiding theft, and so on). As a simple rule of thumb, equipment (also called durable goods, assets, and so on) can be viewed as items that are intended to be used by the NGO to implement its programme across more than one year.

Two general situations exist regarding equipment: (1) the equipment was purchased directly during the programme period; and (2) the NGO already had the equipment before the programme began. A vehicle is a typical example of equipment. Sometimes, vehicles are purchased directly by the project, often towards the beginning of a funding cycle from a donor, for example. Sometimes, an NGO has already purchased or received donated vehicles, but uses them for programme implementation. A building used as an office is another example, where, typically, an NGO may have acquired the building in the past, sometimes many years in the past. A new battery purchased for a laptop computer could also be considered "equipment" because the battery life is intended to be more than one year.

Rather than attempting to justify here an appropriate definition of equipment, two simple criteria can be used to define equipment: (1) the item is intended to be used by the programme over more than one year; and (2) the actual retail price to replace the item is above an "equipment threshold". In many countries, tax policies will provide guidance on what equipment is. Many donors that fund OVC programmes also have their own definitions. For example, individual items purchased for more than $500 is a typical threshold for USAID-funded programmes.

When equipment is rented or leased, the annual rental or lease amount would already be included in the financial report, in which case nothing else is required. These actual payments reflect the annual cost of the equipment to the NGO. Other expenses associated with the equipment, such as maintenance, would also already be included in the financial report.

When equipment is purchased rather than rented, however, the expense is included in the financial report just in the purchase year, but the equipment (e.g., vehicle) is used across multiple years. As a result, expenses are "higher" when the vehicle is purchased, and expenses are "lower" in the years after it is purchased but is being used by the programme.

When evaluating the annual costs of implementing a programme, annualizing equipment purchases, by translating a lump sum payment in one year into a certain number of equal annual payments over multiple years, is an easy way to account for equipment used for programme implementation. Estimating an annual cost equivalent for equipment is also very easy to do with typical spreadsheet programmes that are already used by NGOs for creating their financial reports. Again, no new software is likely to be needed.

NGOs can use any standard "annual payment calculator" to calculate the annual equivalent payment to cover a one-time purchase over a certain time period (e.g., from the purchase year to the end of the project) given a specific discount/interest rate. A "scrap value" can be included if the item continues to have value at the end of the project (e.g., the NGO can sell a used vehicle). For example, the "pmt" function in Excel is easy to use and calculates an annual constant payment that would be required to cover the equipment purchase over a specific time period with a specific interest rate (used to discount future values). For example, if a project purchased a piece of equipment for $10,000 in 2006, and if a five-year working life is assumed, and a 10% annual discount rate is used, the function = pmt(10%, 5, 10,000, 0, 0) yields $2637.97. This is the annualized payment equivalent for the vehicle purchase. If the programme expects to be able to resell the vehicle at the end of the project, for example, for $2500, then the function = pmt(10%, 5, 10,000, -2500, 0) yields $2238.48.

With a 0% discount rate and no resale of the vehicle, the same pmt function yields $2000 (simply the price divided by the number of years). The difference of $637.97 between a 10% and 0% discount rate represents the opportunity cost of funds used to purchase the equipment with a 10% discount rate. While the discount rate should reflect the NGO's opportunity cost of capital (or perhaps social cost of capital), the theory and practice of choosing the right discount rate is less than precise and not addressed here [9]. However, the US government typically tells agencies what discount rate to use when analyzing investment projects (but not all types of projects are required to use the same discount rate). Our view is that financial capital for longer-term investments is obviously scarce for most organizations in sub-Saharan Africa, private companies and NGOs alike. As a result, a positive real discount rate is clearly appropriate to use for programme costing activities. For this analysis, we have chosen to use a 10% discount rate throughout. NGOs and their funding agencies should discuss appropriate discount rates for this type of analysis.

In the example we have used, the purchase occurred in 2006. If the costing analysis was conducted for 2009, the annual payment of $2637.97 based on the 2006 purchase year would have to be inflated to 2009 levels to be included in an analysis for 2009. The annual average consumer price index is logical to use. Such information is typically available from the country's central bank. The International Monetary Fund's *World Economic Outlook Database *(http://www.imf.org/external/pubs/ft/weo/2011/01/weodata/index.aspx) provides easy access to inflation figures for most countries.

### Step 4. Document donations to the NGO for programme implementation

NGOs often receive donated goods and services from other organizations and individuals that are then used for programme implementation (e.g., bednets received from a health project and then distributed to OVC by the NGO). Donations include items provided free of charge to the NGO, as well as items provided at a subsidized price. For example, a nurse might volunteer a day of her time (maybe during annual leave) to work with the OVC programme. If she receives no payment or token of appreciation, her services are free to the project. If she receives something, such as lunch or transportation costs or some small token of appreciation, her services are not free, but very much below what it would cost the project to the hire a nurse for a day. If the donation involves equipment (such as a computer), the market value of the computer would be used as the price used for annualizing equipment purchases addressed in Step 3.

The quantity of donated items should be recorded by the NGO as feasible (for example, as an extra worksheet within its financial report spreadsheet). A market value for many items is typically easy to find so that the costs of such items can be identified. Healthcare products, seeds, fertilizers, clothing, shoes, school supplies and so on are often the types of items that might be donated to NGO programmes (which in turn provide to their programme participants). At a minimum, the quantity of donated items can be recorded so that the market value can be investigated at a later date.

NGO OVC programmes, especially faith-based programmes, typically rely on volunteers for programme implementation. Volunteers receive no salary from the NGO, but sometimes receive some financial payments (small stipends or tokens of appreciation). Information on the number of volunteers involved, the amount of time each contributes to the programme and the services provided as part of programme implementation are usually not well documented. How to obtain better information on volunteers used for programme implementation in a reasonable fashion is beyond the scope of this paper. Until personnel-type records are maintained by NGOs for volunteers contributing to their programmes, however, such information will continue to be based on estimates of varying quality.

By better documentation of the numbers and types of volunteers (for example, local women providing counselling support to other OVC caregivers, extension agents providing advice on agricultural production, medical doctors providing health exams), one goal here is simply to appreciate the importance of such volunteers when considering the expansion or replication of the programme elsewhere. A second goal is to provide a level playing field when considering the costs and outcomes achieved for various OVC programmes. A programme implemented with volunteers might look very inexpensive, but such information would be misleading when considering a replication of the programme elsewhere in the absence of large numbers of volunteers with similar credentials.

### Step 5. Include a portion of NGO organizational costs not attributed to specific programmes

NGOs typically implement multiple programmes. Their OVC programme might be funded through a sub-contract with a US-based organization that has a contract from USAID. A health and sanitation programme might be funded through a UK-based organization with funding from the UK's Department for International Development. An education programme might be funded through a Japanese NGO with funding from Japan International Cooperation Agency..

Each individual programme will likely have a programme-specific financial report. The NGO will also have an annual overall financial report that combines these programmes-specific financial reports and includes additional expenses not attributed to specific projects. For example, a general director or high-level manager might not be accounted for in a specific programme budget. For this step, it is reasonable to allocate a share of the NGO's organizational costs (costs not included elsewhere in specific programmes) to the programme based on the share of the programme's financial costs as a share of all externally funded costs.

### Step 6. Include the results of Steps 3-5 into an expanded cost profile

The final step in this NGO programme costing approach is simply to organize the results from Steps 1-5 into an expanded cost profile that includes the results from Steps 3-5.

## Results

### Introduction to the Community-Based Care for Orphans Program

The CBCO programme provided services to households caring for OVC [18]. These households were often members of the extended family, which is the typical case for OVC in developing countries. As is well recognized, a substantial share of households in developing countries is not able to meet the material and emotional needs of OVC because they are poor. They were poor before assuming responsibility for their charge, and they perhaps became even poorer with the additional person in the household. If the economic situation of these households was adequate, the basic material needs of OVC - food, shelter, clothing, education, healthcare, protection - would be provided by these households and there would be substantially less needs for OVC support programmes.

The core activity within the CBCO programme was the support of village "saving and loan associations" (SLAs). An SLA is comprised of representatives from OVC households (usually a guardian), who form a group (the SLA) and meet regularly (e.g., once a week, twice a month). SLA members make a standard contribution (e.g., 25 Kenyan shillings) at each meeting (savings contributed at SLA meetings are kept in the SLA's lock box). The SLA model, which is a variation on a community-managed micro-finance institution, was developed in the 1990s and been widely adopted around the world [19].

As of 2009, there were 108 SLA groups in the CBCO programme in Kenya (52 with BIDII in Eastern Province and 56 with IDCCS in Nyanza Province), with participation of more than 2500 households. SLA members are typically female, often household heads, who are the primary guardian or "caregiver" for OVC as well as potentially her own children in the household. These households included 7201 OVC in both provinces.

The CBCO programme supported the organization and operation of SLAs in the programme through SLA facilitators. These facilitators attended the regular SLA meetings, provided training on financial management and record keeping (and assisted SLAs as they became familiar with these activities), and provided additional information to the group for income-generating activities. The facilitators were also the conduit through which the CBCO provided supplies to the SLAs (e.g., inputs for income-generating activities) and other services, including additional information and training to SLA members related to business, agriculture and OVC welfare. In many respects, the SLA facilitators acted like agricultural and household extension agents for SLA groups. At times, SLA facilitators would arrange for staff from local government agencies to attend SLA meetings for information exchange.

Each SLA identified two members who served as "mentors" for the CBCO programme. These mentors were responsible for periodically visiting children living in SLA member households. These mentors essentially served as informal social workers in their communities.

The CBCO programme also provided other goods and services directly to households (and sometimes through the SLA). These services included food and nutrition support (provision of seeds, livestock, training on growing kitchen gardens, direct food donations), medical support for OVC in the household, support for school fees for OVC, caregiver training related to child protection and psychosocial support and child protection, and services related to income-generating activities (e.g., information, training). The CBCO programme also provided school-based programmes (youth and kids clubs) through "peer educators" leading after-school programmes (focused on educational support, health and life skills training, and other psychosocial support). SLA members and OVC in the programme did not receive the same "package of services" within or across years.

### Results for Step 1 (access and organize financial reports)

For this analysis, the evaluation year is specifically 1 October 2008 until 30 September 2009, which is the US government fiscal year (FY2009) used for reporting purposes.

**Figure 1:**
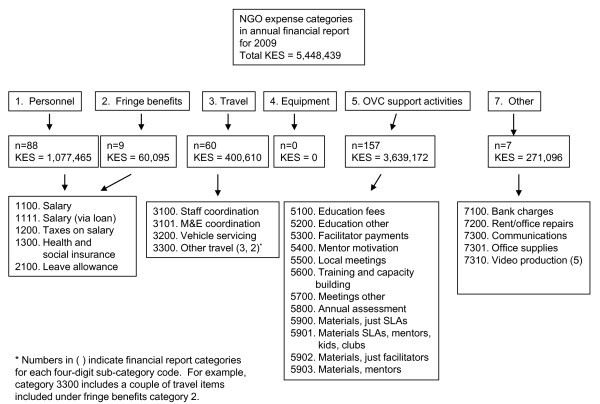
**Structure of BIDII's financial report for 2009**. n = number of itemized expenses in each of the categories in the financial report. The single digit (1-7) financial report categories are taken directly from the NGO's annual financial report. The four-digit financial report sub-category codes were created by the author to group expenses into similar categories.

BIDII's financial report is contained in an Excel spreadsheet with one worksheet for each quarter. These worksheets were then combined into one worksheet for analysis. The worksheet is organized into six major expense report categories, which are listed at the top of Figure 1. Each expense category is coded as 1-7 (no number 6). Each line in the worksheet/dataset is an itemized expense that includes a short description of the expense, the date paid, and an expense number (typically a number written on the original receipt documenting and the payment).

In Figure 1, the number of individual itemized expenses in each financial report category is provided. For example, n = 88 under the personnel category indicates 88 itemized expenses. Total financial expenses for the CBCO programme were 5,448,439 Kenyan shillings (KES) in 2009 ($US75,673 using an average annual exchange rate of KES72 to the US dollar).

After reviewing individual expenses in the financial report database, more detailed sub-categories within each category must be created to facilitate reorganizing information in the financial report into input categories. After reviewing a financial report, consistent groups of itemized expenses are typically easy to identify. For example, in the financial report categories 1 and 2 (personnel and fringe benefits), the 88+9 = 97 itemized expenses fell into five basic sub-categories. At this stage, one extra field (a column in Excel) is added to the financial report and a four-digit financial report sub-category code was created (e.g., 1100 for salary payments, 1300 for health and social insurance payments). The full set of financial category sub-codes with descriptions is provided in Figure 1. Each individual itemized expense in the annual financial report (321 individual line items) is then assigned a sub-category code.

Four typical issues are noted here. First, payments to individuals involved with actual implementation of the project are not always included in the personnel categories. For example, BIDII staff members are included in the financial report categories 1 and 2 as "personnel", while payments to facilitators are included in financial report category 5 (OVC services).

Second, one expense item in the financial report could be one total salary payment for NGO staff for that month (not a specific amount of each individual staff member) or one general payment for social taxes. In general, a detailed breakdown by individuals is not needed or recommended (a costing analysis is not an audit) and would raise multiple issues around confidentiality.

Third, the project purchased no equipment in the year. Issues associated with equipment are discussed in Step 3. And fourth, individual line-item expenses (typically based on an underlying receipt documenting the payment) may combine multiple purchases. For example, financial report sub-category 5901 includes expenses incurred for three reasons (SLA materials, materials for mentors, and materials for kids' clubs organized at local schools) that would be useful to have disaggregated to each specific purpose. When such combined expenses are a relatively small share of total expenses, it is typically not worth the effort to attempt to locate the hard copy of the payment receipt to attempt to breakout into individual items.

### Results for Step 2 (link financial report sub-categories to input cost categories)

Based on the sub-categories in the financial report in Figure 1, Table 2 shows how the expense report sub-categories were allocated to eight major input categories that describe the production structure of the CBCO programme. An extra field (column in Excel), called "cost category code", was added to the financial report. Each itemized expense in the financial report was also given one of the cost category codes.

**Table 2:** Linking financial report categories to key input categories for a cost profile

Costing profile category code	Input category description	Sub-category from financial report	Sub-category description from financial report	Financial report category
1	NGO staff	1100	salary	Personnel

1	NGO staff	1200	taxes on salary	Personnel

1	NGO staff	1300	health and social insurance	Personnel

1	NGO staff	2100	leave allowance	Fringe

2	NGO office	7200	rent or office repairs	Other

3	NGO other office costs/supplies	7100	bank charges	Other

3	NGO other office costs/supplies	7300	communications	Other

3	NGO other office costs/supplies	7301	office supplies	Other

3	NGO other office costs/supplies	7310	Audit fee	Other

3	NGO other office costs/supplies	7310	video production	OVC support

4	NGO travel/meetings/M&E	3100	staff coordination	Travel

4	NGO travel/meetings/M&E	3101	M&E coordination	Travel

4	NGO travel/meetings/M&E	3200	vehicle servicing	Travel

4	NGO travel/meetings/M&E	3300	other travel	Travel

4	NGO travel/meetings/M&E	5500	local meetings and networking	OVC support

4	NGO travel/meetings/M&E	5700	meeting other	OVC support

4	NGO travel/meetings/M&E	5800	annual assessment for CA	OVC support

5	Facilitators (and peer educators)	5300	facilitator payments	OVC support

5	Facilitators (and peer educators)	5902	facilitators	OVC support

6	SLA materials and services	5600	training and capacity building	OVC support

6	SLA materials and services	5900	SLA	OVC support

6	SLA materials and services	5901	materials SLA, mentors, kids' clubs	OVC support

7	Mentors	5400	mentor motivation	OVC support

7	Mentors	5903	mentors	OVC support

8	OVC education expenses	5100	education fees	OVC support

8	OVC education expenses	5200	education other	OVC support

Based on the input categories in Table 2, Table 3 provides a summary of the financial cost structure of the BIDII CBCO programme for FY2009 by input category. Table 3 is the financial "cost profile" for the project. To create Table 3 the financial report was simply sorted by cost category code and then the expenses summed up for each category. Such sorting and summarizing can be done in the financial report electronic file using the same software (e.g., Excel or any other spreadsheet programme). No new software is needed and no separate costing model is needed.

**Table 3:** A cost profile - a summary of BIDII financial expenses reorganized by input cost categories

Input category code	Sub-totals by input category	KES - actual	% total input costs
1	NGO staff	1,112,660	20.40%

2	NGO office	109,200	2.00%

3	NGO other office costs/supplies	207,898	3.80%

4	NGO travel/meetings/M&E	944,711	17.30%

5	Payments for facilitators (and peer educators)	487,340	8.90%

6	Payments for SLA materials and services	836,675	15.40%

7	Payments for mentors	86,700	1.60%

8	Payments for OVC education expenses	1,663,255	30.50%

	Total input costs based on financial report	5,448,439	100.00%

Depending on the size of the project, it sometimes will be useful to disaggregate further the major input categories into more detail as needed. Table 2 provides the logical grouping of sub-categories of inputs that were created in Step 1, which would be used for further disaggregation.

The largest input cost categories in terms of direct financial expenses for the BIDII CBCO programme were direct educational expenses for OVC (30%), BIDII personnel (20%), NGO travel/meetings/monitoring and evaluation costs (17%), and materials for SLAs (15%). Office-based expenses (e.g., office rent and supplies in categories 2 and 3) were a small share of total expenses (< 6%). Table 3 also shows that payments for SLA facilitators and mentors, two key inputs into the production structure of the CBCO programme, accounted for relatively minor shares of total financial expenses (< 9%). Also note that no equipment was purchased during FY2009. We will return to a discussion of cost structure (e.g., is 20% for personnel high, low, typical?) after completing the remaining steps in this costing analysis.

### Results for Step 3 (estimate the annual equivalent payment for programme equipment)

The CBCO programme purchased seven items considered to be equipment during the project, which are listed in Table 4. A service life of five years was assumed, based on the expected programme implementation period. Kenyan inflation (annual average based on consumer price inflation) figures were taken from the International Monetary Fund's *World Economic Outlook Database*.

**Table 4:** BIDII CBCO programme equipment, annualized costs and Kenyan inflation

Asset description	Year purchased	Price (KES)	Service life	Resale value	% rate	Annual to purchase year	Annual 2009
Digital camera	2006	24,500	5	0	10%	6463	8693

Laptop	2006	107,000	5	0	10%	28,226	37,965

Printer	2006	12,500	5	0	10%	3297	4435

Secretarial chair	2008	3399	5	0	10%	897	972

Training board	2008	3500	5	0	10%	923	1000

Training board	2008	3500	5	0	10%	923	1000

Toyota 4X4	2007	2,093,854	5	628,156	10%	449,463	550,790

Total						490,193	604,856

The following consumer price index information was used to inflate purchases from 2006, 2007, and 2008 to 2009 levels: 166.888 for 2006, 183.175 for 2007, 207.171 for 2008, and 224.47 for 2009. Source: International Monetary Fund Financial Statistics (http://www.imf.org/external/data.htm).

The Toyota Land Cruiser was by far the largest equipment purchase, with a price of KES2 million in 2007. This price is actually lower than a typical local price (e.g., in Nairobi) because Christian Aid was able to procure and import the vehicle tax and duty free due to its USAID funding (and US government agreements with the Kenyan government). As a result, this item did not show up directly in BIDII's financial report. If Christian Aid did not receive this subsidy, the purchase price would have been at least 30% to 50% higher. We assume that the vehicle could be resold for 30% of its value after five years. With a 10% discount rate for equipment purchases, the annualized value of all equipment inflated to 2009 values based on Kenyan consumer price inflation is KES604,856. For reference, inflation information for Kenya is also provided in Table 4.

### Results for Step 4 (document donations to the NGO for programme implementation)

Volunteers were a central component of the CBCO programme. BIDII has good information on the number of individuals contributing time for implementing the programme (and different categories of individuals and their activities), but not detailed records on the quantity of time contributed by each individual weekly or monthly or yearly.

As a result, information on the general amount of time that different types of volunteers contributed to the BIDII OVC programme was developed through informal interviews and discussions with programme staff and volunteers. The goal was to understand average amounts of time that various categories of volunteers contributed weekly or monthly. No individually specific information was created in this case. The typical complication here is that some NGO staff might consider themselves to be under paid (and therefore volunteering time to the project), while others considered to be "volunteers" might receive some payments as well (sometimes called motivation, sometimes recognition, and so on).

To consider how to identify volunteers contributing to the programme, all categories of people who contributed time to implementing the programme (paid and unpaid) were identified first. All individuals included as "personnel" in the NGO's financial report (see Table 1 and Figure 1) were considered as employees and therefore not as volunteers.

Besides the NGO staff who implemented the programme (with salary and related payments included in the financial report as personnel and benefits in Figure 1), the CBCO programme relied directly on four groups of individuals for programme implementation:

The *SLA facilitators *provided several types of direct support to SLAs. For example, they assisted with record keeping of the SLA and they delivered programme supplies to the SLA (e.g., the box for savings, locks for the box, materials used for group income-generating activities). Facilitators provide information on income-generating activities that the SLA or individual members might pursue, which might also include organizing for an outside speaker to attend SLA meetings.

*Peer educators *had a similar status as the SLA facilitators within the CBCO programme, but they led the school-based programmes for OVC.

*SLA mentors *were recruited from SLA members to serve essentially as social workers within the project. They conducted direct visits to homes of SLA members for counselling, support and evaluation of OVC caregivers (typically the SLA member) and their children.

The programme relied on a *CBCO committee *in each programme "impact zone". An impact zone is a sub-location in Kenya (government location), and the several SLAs in each sub-location are associated with each CBCO committee.

Table 5 provides estimates of the annual amount of time contributed to the CBCO programme annually. The CBCO operated in six "impact zones". A coordinating committee comprised of 20 members provided oversight support in each zone through a monthly meeting. In addition to general oversight, committee members followed up with individual OVC cases where serious problems were identified. Based on discussions with BIDII, we estimate that each committee member allocated about one day (eight hours) per month to the coordinating committee. With 20 members per zone and six zones, this adds up to about 120 days per month for these committee members.

**Table 5:** Annual labour contribution to the CBCO programme

Volunteer category	Number of volunteers (2009)	Estimated working days spent on project/month per person	Estimated working days spent on project/month whether paid or not	Annual estimate of time (days)
SLA facilitators	12	15	180	2160

Peer educators	12	12	144	1728

Mentors from SLAs	104	7.5	780	9360

Impact zone coordinating committees	120	1	120	1440

Total				14,688

The CBCO programme provided two SLA facilitators and two peer educators for each impact zone (six impact zones), with eight or nine SLAs per impact zone. Based on discussions with programme staff, facilitators and peer educators, facilitators worked about 15 days per month on SLA activities, and peer educators worked about 12 days per month (school-based kids' and youth club activities). Based on these estimates, all facilitators combined contributed 180 days per month of time to the project (2*6*15) while all peer educators combined contributed 144 days per month (2*6*12).

And finally, 102 SLA members contributed to the CBCO programme as mentors. With roughly 30 SLA members in each SLA, each mentor would work with approximately 15 households. Based on discussions with mentors and CBCO programme staff, mentors were estimated to allocate half a day per month to each SLA household. This time includes round trip travel time to the SLA member's home, time for interaction with the SLA member, and time for interaction with children in the SLA household. With 0.5 days per household and 15 households per mentor, this adds up to 7.5 days per month or a total of 780 days per month of mentor time contributed to the CBCO programme.

Because all of these individuals who contribute to the CBCO programme are not employees of BIDII or the programme, records do not actually exist to document actual time contributions of all of these individuals. Since all assumptions are provided in this section, it is easy for a reader to conduct any sensitivity analysis to see how the cost results change if these assumptions on time contributions are changed.

The estimates of annual labour contributions by SLA facilitators, peer educators, mentors and CBCO committee members can be compared with the actual payments made to these individuals to identify an implied daily wage for their efforts. For example, in Table 5, 2160 days are estimated for SLA facilitators annually, and Table 6 shows that these facilitators received KES266,667 in actual payments in 2009 (from the financial report). As a result, the implied daily wage for their services was KES266,667/2160 = KES124 per day ($1.71 using KES72 to the US dollar). Following the same process for mentors, based on actual payments of KES86,000 during the year and an estimate of 9360 days of time, mentors received KES9 per day for their efforts ($0.12 per day).

**Table 6:** The cost of unpaid labour contributions to the BIDII CBCO programme

Category of volunteer	Annual estimate of time (days)	NGO estimate of daily wage	Annual total value at daily wage	Actual payments in financial report	Unpaid annual total (KES) - additional cost of volunteer labour
SLA facilitators	2160	500	1,080,000	266,667	813,333

Peer educators	1728	500	864,000	213,333	650,667

Mentors from SLAs	9360	300	2,808,000	86,000	2,722,000

Impact zone coordinating committee members	1440	500	720,000	0	720,000

Total	14,688		5,472,000	566,000	4,906,000

Table 6 uses information on the quantity of time contributed to the project by each category of volunteer, estimates of a reasonable local wage for such time, and information on actual payments made by the projects to estimate the opportunity cost of time contributed to the project beyond what was paid by the project. For a reasonable daily wage estimate, KES300 (roughly $4 per day) was used for mentors and KES500 per day (roughly $6.7) was used for the other three labour categories. While day labour for agricultural tasks or for tasks that require few skills can be hired for substantially less than these amounts, the amounts used in Table 6 reflect estimates of local wages to hire individuals with skills and capabilities similar to existing volunteers. People in the local community are available to work for a lower daily wage, but the programme relies on a level of skilled labour and responsibilities that are not provided by all individuals in the casual labour market. All information is provided in Tables 4 and 5 for a reader to conduct additional sensitivity analysis of these assumptions.

From Table 6, we estimate that KES4,906,000 of time was contributed by volunteers to the BIDII CBCO programme. Even if the assumptions contained in Table 4 and Table 5 were 100% too high, we would still estimate that KES2,453,000 of time was contributed to the project. This lower figure is still larger than any individual cost category based on financial expenses reported in Table 3.

### Results for Step 5 (include a portion of NGO organizational costs not attributed to specific programmes)

BIDII implemented five separate projects with funding from various sources in 2009. The CBCO programme was the largest, accounting for 51% of BIDII expenses charged directly to one of these five projects. In addition to expenses allocated to these five projects, BIDII incurred additional headquarters costs that were not billed directly to any project (paid for out of its own resources and other donations). We allocated these central costs to the CBCO programme based on its share of total programmes with distinct financial reports (51%), which equalled KES416,549 for 2009.

### Results for Step 6 (include the results of Steps 3-5 in an expanded cost profile)

Based on the additional information developed in Steps 3-5, Table 7 presents the final expanded cost profile for the BIDII CBCO programme. Three additional cost categories (9 = annual asset services, 10 = headquarters, and 11 = impact zone coordinating committees) were added to the expanded cost profile. Table 8 shows the same information reported as shares of total costs within each column of costs. Table 9 summarizes average costs per SLA member and per OVC (in Kenyan shillings and US dollars).

**Table 7:** Expanded cost profile for BIDII CBCO programme

Input category code (Step 2)	Input category description (Step 2)	**Financial expenses (as reported in OVC programme financial report and** Table 3**) (Step 1 and 2)**	Other implementation costs = annualized asset cost + additional cost of volunteer time + BIDII headquarters (Step 3, 4, and 5)	Total = financial expenses + other implementation costs (Step 6)
1	NGO staff	1,112,660	0	1,112,660

2	NGO office	109,200	0	109,200

3	NGO other office costs/supplies	207,898	0	207,898

4	NGO travel/meetings/M&E	944,711	0	944,711

5	Field staff			

	Facilitators	243,670	813,333	1,057,003

	Peer educators	243,670	650,667	894,337

6	SLA materials and services	836,675	0	836,675

7	Mentors	86,700	2,722,000	2,808,700

8	OVC education support	1,663,255	0	1,663,255

9	Annual asset services (2009)	0	604,856	604,856

10	BIDII headquarters contribution	0	416,549	416,549

11	Impact zone coordinating committees	0	720,000	720,000

	Total	5,448,439	5,927,405	11,375,844

**Table 8:** Distribution of costs for BIDII CBCO programme (from Table 7)

Input category code	Input category description	**Financial expenses (as reported in OVC programme financial report and **Table 3**)**	Total = financial expenses + other implementation costs
1	NGO staff	20.4%	9.8%

2	NGO office	2.0%	1.0%

3	NGO other office costs/supplies	3.8%	1.8%

4	NGO travel/meetings/M&E	17.3%	8.3%

5	Facilitators and peer educators		

	Facilitators	4.5%	9.3%

	Peer educators	4.5%	7.9%

6	SLA materials and services	15.4%	7.4%

7	Mentors	1.6%	24.7%

8	OVC education support	30.5%	14.6%

9	Annual asset services (2009)	0.0%	5.3%

10	BIDII headquarters contribution	0.0%	3.7%

11	Impact zone coordinating committees	0.0%	6.3%

	Total	100.0%	100.0%

**Table 9:** BIDII CBCO programme average cost per SLA member and per OVC

Average costs (2009)	Financial expenses		Total costs	
	**KES**	**US$**	**KES**	**US$**

Cost per SLA member	3499	49	7306	101

Cost per OVC in programme	1495	21	3122	43

Average costs were estimated based on the following information for 2009: 1577 SLA members, 3644 OVC, and 72 KES/US$.

The information in Table 7 and Table 8 describes better the production structure of the CBCO programme based on a fuller accounting for all inputs into the programme. For example, NGO personnel costs accounted for 20% of programme financial expenses, but only 10% of total costs. The reason is that the NGO personnel leveraged many thousands of days of volunteer time and support (more than 14,000 days in Table 5). The fundamental role of mentors (about 24% of total costs) is totally missed when considering only financial costs. In sum, total costs in Table 7 are estimated to be about 100% larger than actual expenses included in the programme's financial report due to the quantity of time that mentors, facilitators and peer educators contributed to the programme.

## Discussion

The direct financial cost of implementing BIDII's CBCO programme was $49 per SLA member household and $21 per OVC per year. Most of these costs can be grouped into three key components: direct NGO financial expenses associated with programme implementation, payments to others working essentially as field staff for the programme, but who are not actually employees of the NGOs, and payments for OVC education (fees and supplies). These estimated financial costs from an actual programme are less than 5% of the cost used in recent aggregate modelling exercises mainly because the structure of the CBCO programme did not simply buy and then give numerous sometimes expensive items to large numbers of OVC and their households [20].

The CBCO model of OVC support through SLAs relied on substantial amounts of labour from individuals who were not actual employees of the NGOs. These individuals received varying levels of stipends or gratuities, but the analysis of time contributed to the programme suggests that some significant portion of this time was essentially volunteered. The two key categories of volunteers were "facilitators", who supported SLAs or school-based programmes, and "mentors", who served essentially as social workers for OVC and their guardians. The CBCO programme was designed around the services provided by these individuals. The imputed opportunity cost of this time was $47 per household, so that the estimated total cost of the programme was $101 per household.

While financial costs are based on directly observed itemized expense reports, the information needed for evaluating total costs, especially annualized equivalent payments for equipment and the quantity and values of donations and volunteer time, require various assumptions. Sensitivity analysis is typically used to evaluate how cost estimates change if basic assumptions used in the analysis change (working life of equipment, discount rates, resale values of equipment, quantities of volunteer time, market wages to replace volunteers with paid labour, and so on).

A full discussion of sensitivity analysis is beyond the scope of this paper, but many texts in benefit-cost analyses and economic evaluations cover the topic [7,9,11]. As one example, the estimates of volunteer labour were based on discussions and interviews with programme staff since no detailed records exist of volunteer time and activities. Even if the estimates of volunteer labour costs are 100% too high (i.e., current estimate reduced by 50%), the average cost of the programme for each household would be around $75, which is still substantially higher than direct financial expenses.

In addition to developing a reference cost profile for reporting on the costs of programme implementation, NGOs need guidance on appropriate and feasible ways to document the categories of people contributing to programme implementation that are not considered personnel in financial reports and the amount of time they contribute to the programme (e.g., on a weekly or monthly basis over time).

As a final topic, the six-step method developed in this paper does not provide guidance on apportioning the costs of implementing the overall programme into individual areas of programme support. For example, donors especially are tempted to ask questions like: how much did just the household economic strengthening component cost per OVC, or how much did the food and nutrition support component cost?

Because OVC programmes are inherently joint production processes (some of the same inputs jointly produce multiple outcomes and/or impacts), economic theory provides no guidance on how to apportion costs across multiple outputs. While it is always possible to "divide up" the costs, the information is not especially useful and worse could easily be misleading for improving knowledge on the costs of OVC programmes funded in part through PEPFAR. If and how to apportion costs to individual areas of programme support requires further consideration and is a topic beyond the scope of this paper.

In addition, because the same inputs (e.g., facilitators to support the savings and loan associations in the CBCO programme) will contribute to OVC outcomes across multiple dimensions, it is incorrect to attempt to conduct "cost-effectiveness" analysis by comparing costs for individual programme areas (based on some apportioning of cost logic) with the outcomes achieved just for that programme area. Cost-outcomes analysis, where the costs are the overall programme costs (as developed in this paper) and the outcomes are the set of key outcomes across multiple dimensions, is the most appropriate way to generate useful information for evaluation and comparison of OVC programmes already implemented and to be implemented in the future

## Conclusions

NGOs implementing OVC programmes (and other public health programmes) can organize themselves for data collection and documentation prospectively during the implementation of their OVC programmes so that costing analyses becomes routine practice to inform programme implementation. Information on costs and the structure of costs is required if the costs and outcomes achieved by OVC programmes are ever to be clearly documented and compared across OVC programmes and other types of programmes (prevention, treatment, etc.).

Toward this goal, a set of concrete recommendations are provided here for each step in the method to integrate this costing approach into routine practice of NGOs implementing OVC programmes.

### Recommendations for Step 1 and 2

When setting up financial reporting databases for projects (e.g., an Excel file), the NGO's accounting or financial staff should create their own financial report sub-categories (similar to the four-digit codes in Figure 1) with short descriptions that facilitate understanding the items included in each category. This recommendation is easiest to complete after the first year or at least six months into programme implementation so that the logical sub-categories can be derived based on actual experience. Over time, additional sub-categories may have to be established if the programme structure evolves. The person entering itemized expense information into the NGO's financial report can then add this extra piece of information into the financial report as routine practice.

As much as is reasonable, individual expenses should be disaggregated to be logically allocated to each financial report sub-category to avoid combined categories, such as 5901 in Table 1 in which expenses for three different activities (mentors, SLAs and school-based kids' clubs) were included in the same itemized expense. When one receipt is broken up into multiple expenses in the financial report, the receipt number, date and data would show up multiple times in the financial report, with a portion of the total expense included in that line item with a specific sub-category code. An extra column could be included in the financial report to note that a portion of a total expense receipt is included on multiple lines in the financial report.

As the financial report sub-categories are being developed, financial and programme implementation staff can work together to develop the logical input categories that can be included as an extra field in their financial reporting databases. Each financial report sub-category would be assigned to one of the input categories, so that each individual item in the financial report would have a financial report category (such as 1, 2, 3, 4, 5, 7 in Figure 1), a financial report sub-category code (e.g., the four-digit codes in Figure 1), and an input category assigned to each financial report sub-code (as provided in Table 2). Once the sub-category codes and input category codes are developed and included in the financial reporting database (e.g., the Excel file used for itemized expenses), adding the two extra pieces of information can be done easily as part of entering itemized expenses.

Once the financial expenses are also assigned to specific input categories as part of routine programme practice, the NGO's financial staff can generate their usual financial reports, as well as their cost profiles, easily, quickly and consistently (e.g., as provided in Table 3). No new files, software or models are needed.

### Recommendations for Step 3

The NGO programme, perhaps in collabouration with its funders, should agree at the beginning of the project what will be considered "equipment" and what discount rate should be applied to such purchases. A prerequisite is that the item is used by the project across more than one year. A writing pen, a small stool and a vehicle could all last across multiple years. In addition, some payment level (e.g., $500 or $50 equivalent in local currency) could be chosen. Common sense is required here. If many thousands of pens were purchased at the beginning of a project and then a portion of these pens were supplied each year to OVC for educational purchases, the bulk purchase of pens could be annualized as well. If completely new office furniture was purchased at the beginning of the project, these purchases would logically be annualized as well.

The programme's equipment inventory or register should include the purchase price, including any taxes or duties paid, and the purchase date. This inventory list should include equipment now owned or used by the NGO for project implementation, whether purchased directly by the NGO or provided to the NGO by another organization.

In some countries, bilateral agreements with external donors may allow NGOs to purchase equipment duty and/or value-added tax free. In such cases, the tax status of the equipment should be noted.

NGOs may also include significant training for their staff or programme participants, especially during the beginning of their programmes. If such training is viewed as investment in human capital, these costs could be annualized as well. For example, if an analysis of programmes costs in 2009 was being completed, it would be appropriate to include the annual equivalent for training costs incurred in previous years.

NGOs may also include significant start-up costs, typically in Year 1 of the programme. These start-up costs could also be viewed as an investment in the programme, so that these costs could be annualized as well. For example, if an analysis of programme costs in 2009 was being completed, it would be appropriate to include the annual equivalent of the start-up costs incurred in Year 1. As with equipment, the basic logic is that start-up costs are incurred, but provide services to the programme over the life of the programme.

### Recommendations for Step 4

NGOs should add an extra sheet into their financial report that accounts for the quantity (physical units), market price of the items if available (e.g., retail price), and/or value (price times the number of units) of items donated or provided at subsidized prices to the NGO. While NGOs will typically have a record somewhere documenting such donations, it is often difficult for NGO staff to remember all such donations, especially in previous years. Rather than having to search for such documentation, including this information within the programme's overall financial report (for example, as a separate worksheet in an Excel file) is easy and useful to do.

Accounting for the quantity of volunteer time used by the programme remains problematic. NGOs should discuss reasonable and not onerous approaches for documenting who is volunteering how much time to the programme. A solution to this problem is beyond the scope of this paper, but a range of options (less or more data intensive) are likely to make sense, depending on the situation.

### Recommendations for Step 6

Create the financial cost profile and the expanded cost profile based on Step 6 when developing the annual financial report.

Clearly document all assumptions used in Steps 3-5 so that the implications of these assumptions on the expanded cost profile can be easily evaluated.

In conclusion, a cost analysis using the approach outlined in this paper simply identifies the cost and cost structure, based on the major input categories, of an OVC programme. In addition to direct financial expenses, the contributions of volunteers, equipment and other possible assets are incorporated to provide a fuller picture of the how the programme was implemented. In parallel with demands for more reporting on the costs of implementing OVC programmes, better information is needed on the impacts or benefits of such programmes. The demand for such information is already incorporated, for example, into USAID's relatively new evaluation policy [21]. It is too early to tell if the new policy will generate such information.

## Competing interests

The authors declare that they have no competing interests.

## Authors' contributions

BL developed the method, completed data analysis, and drafted and revised the manuscript.

NW contributed to developing the method, developing data for the BIDII case study, and revising the report. Both authors have read and approved the final version of this manuscript.
